# Determination of Acid Dissociation Constants (pK_*a*_) of Bicyclic Thiohydantoin-Pyrrolidine Compounds in 20% Ethanol-Water Hydroorganic Solvent

**DOI:** 10.1155/2014/634194

**Published:** 2014-03-30

**Authors:** Yahya Nural, H. Ali Döndaş, Hayati Sarı, Hasan Atabey, Samet Belveren, Müge Gemili

**Affiliations:** ^1^Department of Chemistry, Faculty of Pharmacy, Mersin University, 33169 Mersin, Turkey; ^2^Advanced Technology Research & Application Center, Mersin University, Yenisehir, 33343 Mersin, Turkey; ^3^Department of Chemistry, Faculty of Science and Arts, Gaziosmanpasa University, 60250 Tokat, Turkey

## Abstract

The acid dissociation constants of potential bioactive fused ring thiohydantoin-pyrrolidine compounds were determined by potentiometric titration in 20% (v/v) ethanol-water mixed at 25 ± 0.1°C, at an ionic background of 0.1 mol/L of NaCl using the HYPERQUAD computer program. Proton affinities of potential donor atoms of the ligands were calculated by AM1 and PM3 semiempiric methods. We found, potentiometrically, three different acid dissociation constants for **1a–f**. We suggest that these acid dissociation constants are related to the carboxyl, enol, and amino groups.

## 1. Introduction

Acid dissociation constants are important physicochemical parameters, which can provide critical information about drug properties such as solubility, lipophilicity, acidity [1–3], transport behavior, bonding to receptors [2–4], and permeability [5]. Hence, the relationship between the acid dissociation constants and structure in drug design studies is important [2, 3, 6]. Acid dissociation constants are also important parameters for the selection of the optimum conditions in the development of analytical methods [5, 7] and provide information about the stereochemical and conformational structures of active centers of enzymes [8]. Acid dissociation constants are determined by various methods such as potentiometric [5, 9], spectroscopic [6], and electrophoretic methods [2, 10].

Thiohydantoins [11, 12] are potential bioactive heterocyclic compounds with pharmaceutical and agricultural applications [13] due to their antimicrobial [14], antiviral [15], anti-inflammatory [13], antitumor [16, 17], and antiandrogenic properties [18]. In addition, 2-thiohydantoins are useful intermediates in the synthesis of natural products [19, 20]. Pyrrolidines and their derivatives are also present in many natural products [21] and biologically important synthetic compounds [22–24].

In the previous work [25], we have reported the synthesis and structural determination of a series polysubstituted bicyclic thiohydantoins fused to pyrrolidines. In this study, potentiometric and theoretical studies of some polysubstituted thiohydantoin-pyrrolidine fused ring systems are reported.

## 2. Experimental

### 2.1. Apparatus and Materials

All reagents having analytical grade were used without further purification. Sodium hydroxide (Merck), potassium hydrogen phthalate (Fluka), and sodium tetraborate (Fluka) were dried at 110°C before use and 0.05 mol/kg potassium hydrogen phthalate and 0.01 mol/kg sodium tetraborate solutions were prepared for calibration of the electrode systems. Solution of ligands 1.10^−3 ^mol/L in 20% (v/v) ethanol-water, 0.025 mol/L NaOH, and 0.1 mol/L HCl (J. T. Baker) and 1.0 mol/L NaCl (Riedel-de Haën) stock solution were prepared. Deionized water with a resistance of 18.2 MΩ·cm was obtained with an aquaMAX-Ultra water purification system (Young Lin Inst.).

pH-Metric titrations were performed using the Molspin pH meter with an Orion 8102BNUWP ROSS ultracombination pH electrode. The temperature in the double-wall glass titration vessel was kept at 25.0 ± 0.1°C and controlled using a thermostat (DIGITERM 100, SELECTA). During the titration, the vessel solution was stirred. The combination pH electrode was calibrated by the buffer solutions of pH 4.005 (potassium hydrogen phthalate) and 9.018 (sodium tetraborate) at 25.0 (±0.1)°C according to the instructions of the Molspin Manual [26]. An automatic microburette was connected to a Molspin pH-mV-meter.

During the titration, nitrogen (99.9%) was purged through the titration vessel. Acidity and stability constants were computed using the HYPERQUAD computer program [27].

### 2.2. Procedure

The potentiometric titrations were carried out using Molspin pH meter with an Orion 8102BNUWP ROSS ultracombined electrode. The titration temperature was adjusted at 25.0 ± 0.1°C and controlled by a thermostat (DIGITERM 100, SELECTA). The double-wall glass titration vessel was used and the titration solution was magnetically stirred. Before and after each titration, the double-wall glass titration vessel was rinsed with distilled water and dried with a piece of tissue. The titration vessel was capped by a lid, which contained three holes for the electrode, glass tubing for nitrogen purging, and plastic tubing for alkali from the burette. Air bubbles were excluded while dropping with alkali solution. The syringe was rinsed with deionized water and at least three times with the alkali before filling with the alkali solution. The acid dissociation constants were determined by titrating 50.00 mL of ligands with standardized NaOH solution and three titrations were performed for each ligand. HYPERQUAD computer program was used for the calculation of acidity and stability constants. Standard deviations quoted only refer to random errors. 130 of the titration data were obtained for each experiment. Volume increment of the NaOH solution was 0.03 mL. The pKw value as, defined for the aqueous system was obtained as 13.98 at the ionic strength employed.

## 3. Results and Discussion

**Figure 1:**
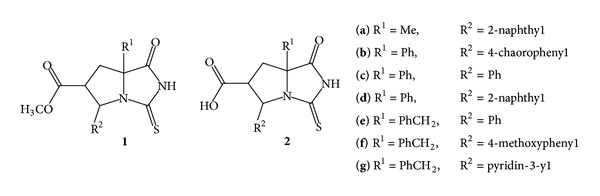
Methyl ester of thiohydantoin-pyrrolidine compounds (**1a–g**) and their hydrolysis products in acidic media (**2a–g**).

Acid dissociation constants of the thiohydantoin-pyrrolidine compounds containing methyl ester group as a substituent (**1a–g**), prepared according to the literature method [25], were determined potentiometrically at 25.0 (±0.1)°C in a 20% (v/v) ethanol-water mixture. The solutions were prepared in acidic media and the ionic strength was kept constant at 0.1. HYPERQUAD is one of latest developed computer programmes for calculating equilibrium constants from potentiometric and spectrophotometric data [27]. In this respect, the programme is quite useful. In this study, it was used for calculating acid dissociation constants of ligands from potentiometric data. We found potentiometrically three different pK_*a*_ values for each compound, except for** 1g** which had four acid dissociation constants due to the presence of the pyridine group. For the thiohydantoin-pyrrolidine fused ring systems (**1a–g**), pK_*a*1_, pK_*a*2_, and pK_*a*3_ values were found in a range of 3.21 ± 0.02–3.80 ± 0.05, 8.13 ± 0.01–8.66 ± 0.02, and 10.87 ± 0.01–12.70 ± 0.05, respectively. pK_*a*1_ values (a range of 3.21 ± 0.02–3.80 ± 0.05) arise from hydrolysis of the methyl ester in acidic aqueous solution. In acidic solution, the weak nucleophile water attacks carbonyl carbon atom, having protonated oxygen atom; then carboxylic acid derivatives and alcohol groups are formed [28]. Similarly, carboxylic acid derivatives of the thiohydantoin-pyrrolidine compounds (**2a–g**) are formed, after hydrolysis of methyl ester groups of thiohydantoin-pyrrolidine compounds (**1a–g**) (Figure 1). Despite all this, according to protonation of the thiohydantoin-pyrrolidine fused ring systems in acidic solution, the thiohydantoin-pyrrolidine fused ring systems containing carboxylic acid group (**2a–f**) may have four acidic centers as carboxyl, enthiol, enol, and amino group. As a result of theoretical and experimental calculations, we suggest that pK_*a*1_ value is related to the carboxyl group; pK_*a*2_ and pK_*a*3_ values are related to enol and amino group, respectively.

Acid dissociation constants were potentiometrically obtained from several series of independent measurements. Three deprotonated species that formulated as LH_3_, LH_2_, and LH were observed during titration processes. The deprotonation equilibrium is as seen in the following equations (charges are omitted for simplicity):

(1) $$\begin{array}{c} {\mathrm{LH}}_{n}⇌{\mathrm{LH}}_{n-1}+\text{H} \end{array}$$

and the deprotonation constants (K_*n*_) are given as

(2) $$\begin{array}{c} {\text{K}}_{n}=\frac{\left[{\mathrm{LH}}_{n-1}\right]\left[\text{H}\right]}{\left[{\mathrm{LH}}_{n}\right]}. \end{array}$$

**Figure 2:**
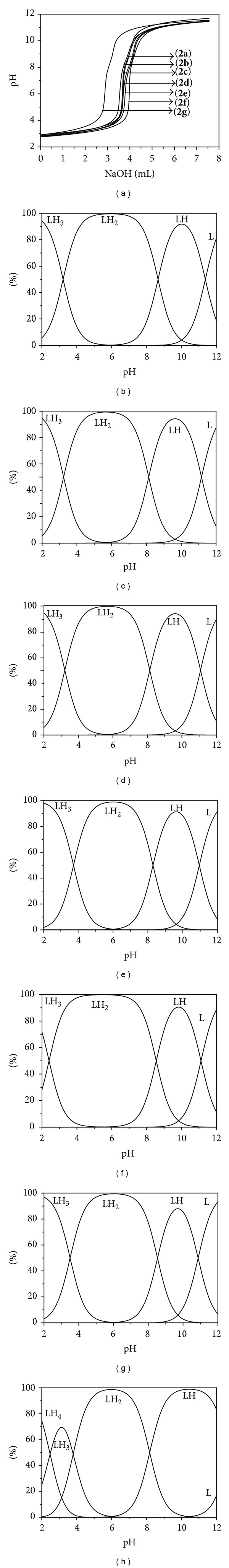
Potentiometric titration curves (a) and distribution curves of ligands ((b)** 2a** (c)** 2b** (d)** 2c** (e)** 2d** (f)** 2e** (g)** 2f** (h)** 2g**) (20% (v/v) ethanol-water mixed, 25.0 ± 0.1°C, *I* = 0.1 mol/L by NaCl).

All species have broad protonation space between pH 2 and 12. When pH increases, the protonated ligand losses protons and it is converted to the other forms. The titration curves of the ligands (**2a–g**) and the distribution curves of species H in 20% (v/v) ethanol-water mixed are shown in Figure 2.

Acid dissociation constants of ligands were computed from data which were obtained by potentiometric titrations using HYPERQUAD computer program. These acid dissociation constants are given in Table 1.

Theoretical calculations were made in order to examine the structure of the species and to determine protonation order of nitrogen, oxygen, and sulphur atoms in the ligands. The formation heats (*H*_*f*_) and the total energies (TE) of the ligands and monoprotonated species were calculated by semiempirical AM1 and PM3 methods. In addition, the proton affinity of each nitrogen, oxygen, and sulphur atom (PA) in the ligands was found using formation heats in the following equation and given in Table 2:

(3) $$\begin{array}{c} \text{PA}=367.2+\Delta H_{f}^{{}^{\circ}}\left(\text{B}\right)-\Delta H_{f}^{{}^{\circ}}\left({\text{BH}}^{+}\right), \end{array}$$

where PA is the proton affinity of B types, Δ*H*_*f*_°  (B) is the formation heat of B molecule, Δ*H*_*f*_° (BH^+^) is the formation heat of BH^+^ molecule, and 367.2 is the formation heat of H^+^ [29].

Proton affinity gives information about protonation order. Since the nitrogen, oxygen, and sulphur atoms having the highest PA are 2S in ligand, in other ways, 2S is more basic than other nitrogen and oxygen groups. Thus, the first protonated atom is 2S in this ligand. According to the calculated results (TE, *H*_*f*_, and PA), protonation orders of atoms in the ligand are 3S, 4N, 1O, and 5O, but dissociation orders of atoms in the H are 5O, 1O, 4N, and 3S. There might be differences in these orders due to forming intermolecular hydrogen bonding and forming acid thiol and enol group because of tautomerism in the ligand. It is assumed that high affinity causes the protonation of the sulfur and oxygen atoms on ligand and increases the mobility of its *π*-electrons. Consequently, sulfur and oxygen atoms of thiohydantoin begin to be hydrolyzed at appreciable rates in the solutions more alkaline than pH 9 [30, 31].

According to the electronic delocalisation, which is enhanced upon deprotonation, the ligand is very versatile, because thione and ketone forms were easily transformed into thiol and enol forms depending on pH [32, 33]. Therefore, the ligand (2H^3+^) has got four ionizable groups as thiol, enol, amino, and carboxyl in ligands. In other ways, the potential coordinating sites are sulphur atom of the thiol group, nitrogen atoms of the amino group, and oxygen atoms of the carboxyl group and hydroxyl group. But, due to intermolecular hydrogen bonding between the neighbouring nitrogen atoms and the basic nature of the sulphur atom, the dissociation of the thiol group takes place at a very high pH level. Thus acid dissociation constant of thiol group was not determined in our experimental conditions.

## 4. Conclusion

In conclusion, the acid dissociation constants of thiohydantoin-pyrrolidine fused derivatives, which constitute important information for future studies in this area, were determined potentiometrically and the results were interpreted with the HYPERQUAD computer program. The determination of the acid dissociation constants was performed in 20% (v/v) ethanol-water hydroorganic solvent at 25 ± 0.1°C, at an ionic background of 0.1 mol/L of NaCl. Under these conditions, three different pK_*a*_ values for** 1a–f** were calculated. In addition, theoretical calculations were carried out by AM1 and PM3 semiempiric methods.

## Figures and Tables

**Table 1:** Acid dissociation constants (pK_*a*_) of ligands (20% (v/v) ethanol-water mixed, 25.0 ± 0.1°C, *I* = 0.1 mol/L by NaCl) (log_10_⁡*β* is cumulative acid dissociation constants).

Ligand	Species	log_10_⁡*β*	pK_*a*_ Values
**2a**	LH_3_	11.37 ± 0.02	3.22 ± 0.01
	LH_2_	20.03 ± 0.03	8.66 ± 0.02
	LH	23.25 ± 0.03	11.37 ± 0.01

**2b**	LH_3_	11.16 ± 0.04	3.24 ± 0.01
	LH_2_	19.29 ± 0.05	8.13 ± 0.01
	LH	22.53 ± 0.05	11.16 ± 0.03

**2c**	LH_3_	11.08 ± 0.02	3.21 ± 0.02
	LH_2_	19.23 ± 0.03	8.15 ± 0.01
	LH	22.44 ± 0.03	11.08 ± 0.02

**2d**	LH_3_	10.95 ± 0.08	3.74 ± 0.04
	LH_2_	19.24 ± 0.09	8.29 ± 0.03
	LH	22.98 ± 0.09	10.95 ± 0.05

**2e**	LH_3_	11.12 ± 0.04	3.40 ± 0.02
	LH_2_	19.67 ± 0.04	8.55 ± 0.02
	LH	23.07 ± 0.05	11.12 ± 0.04

**2f**	LH_3_	10.87 ± 0.05	3.50 ± 0.03
	LH_2_	19.40 ± 0.07	8.53 ± 0.03
	LH	22.90 ± 0.08	10.87 ± 0.01

**2g**	LH_4_	12.70 ± 0.11	2.48 ± 0.07
	LH_3_	20.88 ± 0.13	3.80 ± 0.05
	LH_2_	24.68 ± 0.10	8.18 ± 0.06
	LH	27.16 ± 0.09	12.70 ± 0.05

**Table 2:** The calculated *H*_*f*_, TE and PA values with AM1 and PM3 methods for ligand and its monoprotonated forms.

Species	AM1		
	TE (kcal/mol)	*H*_*f*_ (kcal/mol)	PA
H	−59403.18	−87.83	—
1 O–H^+^	−59545.75	84.41	194.96
2 S–H^+^	−59560.21	69.95	209.42
3 N–H^+^	−59546.16	83.99	195.38
4 O–H^+^	−59513.86	116.30	163.07

Species	PM3		
	TE (kcal/mol)	*H*_*f*_ (kcal/mol)	PA

H	−54070.31	−101.20	—
1 O–H^+^	−54238.82	83.77	182.23
2 S–H^+^	−54265.61	57.08	208.92
3 N–H^+^	−54242.16	80.53	185.47
4 O–H^+^	−54219.39	103.29	167.71
